# Study of Thermal Effect on the Mechanical Properties of Nylon 610 Nanocomposites with Graphite Flakes That Have Undergone Supercritical Water Treatment at Different Temperatures

**DOI:** 10.3390/polym14245494

**Published:** 2022-12-15

**Authors:** Jun-Ven Lim, Soo-Tueen Bee, Lee Tin Sin, Chantara Thevy Ratnam, Soo-Ling Bee

**Affiliations:** 1 Department of Mechanical and Material Engineering, Lee Kong Chian Faculty of Engineering and Science, Universiti Tunku Abdul Rahman, Jalan Sungai Long, Bandar Sungai Long, Cheras, Kajang 43000, Selangor, Malaysia; 2 Department of Chemical Engineering, Lee Kong Chian Faculty of Engineering and Science, Universiti Tunku Abdul Rahman, Jalan Sungai Long, Bandar Sungai Long, Cheras, Kajang 43000, Selangor, Malaysia; 3 Radiation Processing Technology Division, Malaysian Nuclear Agency, Bangi, Kajang 43000, Selangor, Malaysia; 4 School of Materials and Mineral Resources Engineering, Engineering Campus, Universiti Sains, Bukit Panchor, Nibong Tebal, Malaysia, Bukit Panchor, Nibong Tebal 14300, Pulau Pinang, Malaysia

**Keywords:** mechanical properties, graphite flakes, polymer nanocomposites, supercritical water treatment

## Abstract

This study investigates the thermal effect of supercritical water treatment at different temperatures (150, 175, 200 °C) and semi-vacuum state (−0.08 MPa) on graphite flakes which are then incorporated into nylon 610. The treatment is deemed to increase the surface activity of nanofillers through the formation of oxygen-containing functional groups. X-ray diffraction (XRD) analysis indicated that the crystal structure of the flakes remained similar before and after supercritical water treatment. Fourier transform infrared spectroscopy (FTIR) also showed the presence of hydrogen bonding between the flakes and the polymer matrix through the appearance of amide bands. The intensity of the amide peaks is higher for nanocomposites with treated flakes than untreated ones. Furthermore, scanning electron microscopy (SEM) showed that at higher wt%, aggregation will occur, which leads to a weakening in physical properties. The tensile strength of nanocomposites with treated flakes decreased with increasing wt%, while those with untreated flakes increased with increasing wt%. Young’s modulus of all the nanocomposites generally increased with increasing wt%. The highest tensile strength obtained is 967.02 kPa, while that of neat nylon 610 is 492.09 kPa. This enhancement in mechanical properties can be attributed to the intact structure of the graphite flakes and the interaction between the flakes and the nylon 610 matrix. A higher temperature of water treatment was discovered to cause higher oxidation levels on surface of the nanofillers but would result in some structural damage. The optimum nylon 610 nanocomposite synthesized was the one that was incorporated with 1.5 wt% graphite flakes treated at 150 °C and −0.08 MPa, as it has the highest tensile strength.

## 1. Introduction

Polymers are composed of large molecules built up by the repetitive bonding of monomers. One such polymer is nylon, which is a synthetic polymer made up of polyamides; it being a semi-crystalline thermoplastic also allows it to be processed into many different forms, such as fibers and films. This ease of processing has resulted in it being used in many engineering and commercial applications. However, occasionally, nylon polymers are incorporated with additives such as nanofillers to create nanocomposites in order to enhance their properties to suit usage in harsh environments. One type of nanofiller that is employed in the synthesis of these composites is carbon nanofillers, such as carbon nanotubes [1], foliated graphite [2] and graphene oxides [3], as they have superb mechanical, electrical and thermal properties. Research has also shown that these graphite-based nanocomposites saw large improvements in their mechanical and electrical properties [4]. As a result, in the past decade, these nanostructures were widely studied as part of a novel generation of composite materials. In this research, graphite flakes were chosen to be used as nanofillers to be incorporated in nylon 610, as they are relatively cheap and widely available. Graphite flakes also remain as one of the few carbon nanofillers that can be sufficiently produced at the rate necessary for applications in polymer composite materials and nanostructures [5].

However, the degree of enhancement obtained after adding the carbon nanofillers to the polymer matrix is generally lower than anticipated. This is due to two reasons: the first being the weak interface bonding between the polymer matrix and these carbon nanofillers, and the second being the poor dispersion of the nanofillers due to their tendency to agglomerate in the polymer matrix [6,7]. Thus, in this research, one of the aims is the functionalization of graphite flakes through supercritical water treatment in an attempt to overcome these flaws, as functionalization of carbon nanofillers is known to minimize agglomeration and enhance the dispersion of nanofillers in solvents [8]. Functionalization via supercritical water treatment is attained when the surface of the graphite flakes are modified by the formation of oxygen-containing functional, such as hydroxyl, carbonyl or carboxylic acid, groups. The effect of supercritical water treatment on carbon nanofillers is also the subject of growing interest in nanocomposites research due to its effect in preparing polymer composites. This is due to the many special properties supercritical water has, such as superb oxidation, solubility and diffusion abilities [9]. The fluid also possesses both characteristics of gas (high diffusivity) and liquid (high collision rates). While there are different supercritical solvents that can be used in the functionalization of carbon nanofillers, such as supercritical carbon dioxide (CO_2_), supercritical water is chosen, as water is readily available, inexpensive, non-toxic and safe to handle. Supercritical water treatment is generally performed by adding distilled water and the carbon nanomaterial into a reaction vessel. The vessel or reactor is then heated to supercritical temperatures of 373 °C and the pressure is raised to 22.1 MPa. Thus, this research into supercritical water treatment could help improve the unsatisfactory attributes of the original polymer and produce high performance polymer nanocomposites that can be applied in harsh environments.

Additionally, within this research, three different set of temperatures were used for the supercritical water treatment of the graphite flakes (150 °C, 175 °C, 200 °C) so as to determine the results of the different thermal effects on the nanofillers. Additionally, while there are a few articles that have reported on the results of treating carbon nanofillers using supercritical water, the novelty of this research is that even less research has examined the effects of treating the nanofillers at different temperatures and pressures from the supercritical condition. In the present work, we aim to analyze the effect of the treated and untreated graphite flakes in the nylon 610 matrix, along with the mechanical properties and microstructures of the nanocomposites.

## 2. Materials and Methodology

### 2.1. Materials

The nanofillers that were employed in this research are graphite flakes (which have a particle diameter of 2–10 nm), which were manufactured by Qingdao Tiansheng Graphite Co., Ltd. in Qingdao, China. The graphite flakes were also the only reinforcing filler used in this research. The purity of the flakes is labeled as ≥99%. The chemical reactants used to synthesize the polymer base of nylon 610 were hexamethylenediamine (HMDA) and sebacoyl chloride. Both of these were manufactured by Merck Sdn Bhd in Subang Jaya, Malaysia, and had a purity of ≥99%. The hexane that was used as a base to dissolve sebacoyl chloride, along with the sodium hydroxide (NaOH) used to neutralize the hydrogen chloride that formed after the interfacial polymerization reaction were also manufactured by Merck Sdn Bhd. The purities of the hexane and NaOH were also ≥99%.

### 2.2. Preparation of Nylon 610/Graphite Flakes Nanocomposites

**Figure 1:**
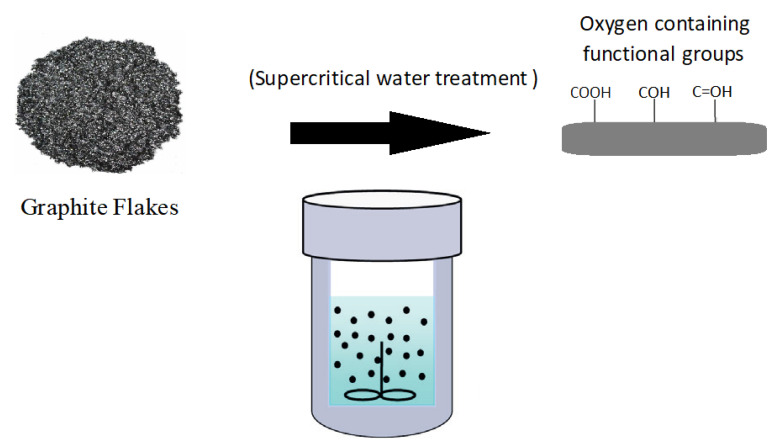
Supercritical Water Treatment of Graphite Flakes.

Firstly, to prepare for the supercritical water treatment, the required amount of graphite flakes and 100 mL of distilled water were added to a 250 mL beaker. A homogenizer was used to stir the mixture for 10 min in order to evenly distribute the flakes in the water. Next, the graphite flake and distilled water mixture is placed inside a vacuum oven. The pressure in the vacuum oven is kept at a semi-vacuum state of −0.08 MPa, while the temperature is maintained at 200 °C for a length of 30 min. The purpose of using this low pressure is to help lower the boiling point of the water. After the supercritical water treatment is finished, the pressure and temperature are reduced to atmospheric pressure and room temperature, respectively. The supercritical water treatment of graphite flakes is repeated 5 times for the different loading levels (0.5, 1.5, 2.0, 3.5 and 4.5 wt%) that will be added to the nylon 610. The reason behind adding a relatively small amount of nanofillers (below 5 wt%) to the polymer matrix is because only a small amount is required before noticeable improvements to the properties of the nanocomposites will surface [10]. The supercritical water treatment is also repeated using different temperatures of 175 °C and 150 °C with the same 5 graphite loadings. The process of supercritical water treatment is shown in Figure 1.

The synthesis of nylon 610/graphite flake composites via interfacial polymerization was accomplished by first creating an aqueous phase comprised of 6.0 g (7.143 mL, 51.63 mmol) of HMDA and 2.0 g (0.5 M, 50 mmol) of NaOH added to 100 mL of distilled water with the supercritical water-treated graphite flakes mixed inside. Over this solution, the organic phase, which is made of 2.0 mL (2.24 g, 9.367 mmol) sebacoyl chloride in 100 mL hexane, is carefully poured on top of it, as it is less dense than the aqueous phase. The polymerization reaction began immediately upon addition of the organic phase dispersion and a film of nylon 610/graphite flake composite is formed at the liquid interface. The film is grasped with tweezers and raised as a rope of continuously forming nylon 610/treated graphite flake film on a glass rod roller. The nylon 610/treated graphite flake composite is then washed well with distilled water and then soaked in distilled water for 30 min to get rid of any remaining chemicals. The damp sample was then dried in an oven at 60 °C for 24 h. These steps are then repeated for the different graphite loadings. Furthermore, to discern the effect of supercritical water treatment on the graphite flakes, the above steps are also repeated by using untreated graphite flakes or graphite flakes in its original state. Finally, neat nylon 610 was also synthesized, using the methods above, in order to act as a control.

### 2.3. Characterization Testing

#### 2.3.1. Tensile Testing

The tensile strength and Young’s Modulus of the nylon 610/graphite flake nanocomposites were determined using a Shidmazu Servopulser machine. The nanocomposites were cut into rectangular strips with a uniform length of 10 cm, while the width and thickness of the composites were measured using a Vernier caliper before proceeding with the tensile testing. The tensile tests were conducted using a gauge length of 5 cm, a strain rate of 5 mm/min and with a cell load of 1 kN. The tensile strength and Young’s modulus of each sample were taken as an average of 5 specimens.

#### 2.3.2. X-Ray Diffraction (XRD) Analysis

XRD data are obtained using a Shidmazu XRD-6000 Diffractometer that uses CuKα radiation. Firstly, the sample is placed in an aluminum plate holder. Then, the X-ray tubes will generate the CuKα radiation, which has a wavelength of 1.542 Ǻ, while the Rotational Sample Stage has a measuring angle range of 2θ = 5° to 40° and has a scanning rate of 1.2° min^−1^. The operating current and acceleration voltage of the Cu-Kα radiation generator were set at 30 mA and 40 kV, respectively. The interlayer spacing or d-spacing, *d*, of crystallites was calculated using the Bragg’s equation, which is shown in Equation (1) [11]. Additionally, the inter-chain separation, *R*, of crystallites was determined by using the Klug and Alexander equation, as shown in Equation (2) [11]. The XRD test was performed to analyze the dispersion of the graphite flakes within the nylon 610 matrix at 40 kV voltage.

(1) $$d=\frac{\lambda }{2\;\sin\theta }$$

(2) $$R=\frac{5\lambda }{8\sin\theta }$$

where *λ* is 1.542 Å and *θ* is the diffraction angle in the XRD spectra.

#### 2.3.3. Fourier Transform Infrared Spectroscopy (FTIR) Analysis

For this research, FTIR spectroscopy was performed using a Nicolet iS 10 FTIR Spectrometer in order to determine the presence of certain functional groups and chemical bonds in the graphite flake/nylon 610 nanocomposites. The FTIR spectra of the samples were recorded ranging from the band region of 4000 cm^−1^ to 400 cm^−1^.

#### 2.3.4. Scanning Electron Microscopy Analysis (SEM)

SEM is used to determine the surface morphology of the nylon 610 nanocomposite samples and graphite flakes (both treated and untreated). The nanocomposite specimens were broken in half and the fractured half was then placed face up on top of the specimen stub (with a diameter of 10 mm) using carbon tape. The mounted samples were then coated in a layer of gold and palladium using an EMITECH SC7620 sputter coater to prevent charging of the samples. The samples were investigated using a Hitachi S-3400N SEM machine with 15 kV of operating voltage. The surface of the specimen is recorded at a magnification of 1000 times.

## 3. Results and Discussion

### 3.1. Mechanical Properties

#### 3.1.1. Tensile Strength

**Figure 2:**
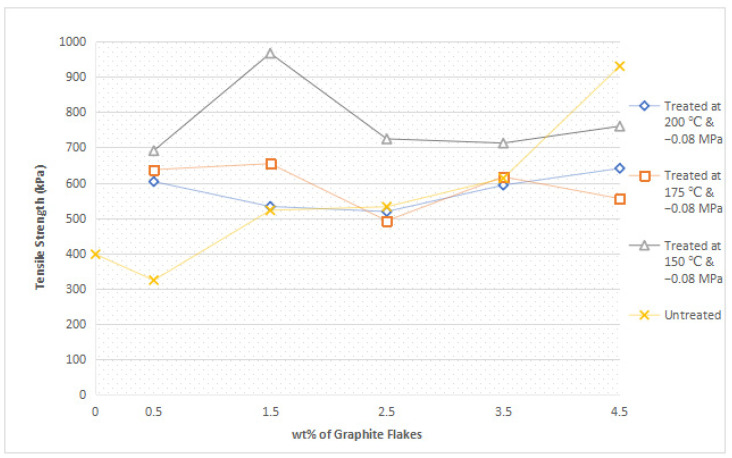
Average Tensile Strength of All the Nylon 610/Graphite Flake Nanocomposites.

**Figure 3:**
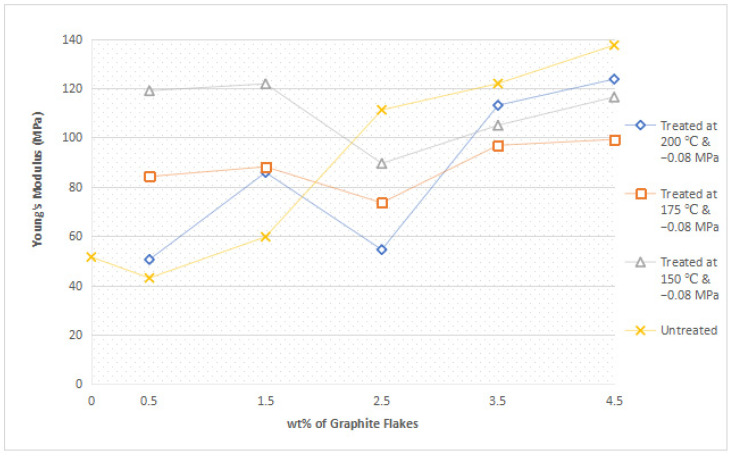
Average Young’s Modulus of All the Nylon 610/Graphite Flake Nanocomposites.

In this research, different loading levels of graphite flakes ranging from 0.5 wt% to 4.5 wt% were incorporated into nylon 610; and the tensile strength and Young’s Modulus of these nylon 610 nanocomposites are shown in Figure 2 and Figure 3, respectively. The data for the mechanical properties are also recorded in Table 1, while the bar charts for the mechanical properties with error bars are shown in Figures S1–S8. As shown in Figure 2, for nanocomposites with graphite flakes treated at 200 °C and −0.08 MPa, the tensile strength of the composites first decreases with an increase in the loading level of treated flakes, eventually reaching a minimum at 2.5 wt% (518.18 kPa), before gradually increasing and reaching its highest at 4.5 wt% (640.76 kPa); however, when compared with the rest of the nanocomposites, the addition of the flakes treated at 200 °C and −0.08 MPa was shown to have relatively little effect on tensile strength, as the tensile strengths are similar to one another regardless of the loading level. On the other hand, the incorporation of flakes treated at 175 °C and 150 °C resulted in the tensile strength generally decreasing with increasing loading level after reaching a maximum at 1.5 wt%. For untreated graphite flakes, however, tensile strength generally increases with increasing wt%, eventually reaching its highest point at 4.5 wt% (930.49 kPa), similar to the nanocomposites with flakes treated at 200 °C.

The decrease in tensile strength for nanocomposites with graphite flakes treated at 175 °C and 150 °C from 1.5 wt% onwards is due to the increasing amounts of flakes within the nylon 610 matrix, which gives rise to agglomerates of the nanofiller particles. The formation of aggregates at higher wt% can be seen in Section 3.4. This is seen when it was the nanocomposite incorporated with 1.5 wt% flakes (a low loading level) treated at 150 °C that possessed the highest tensile strength (967.02 kPa) out of all the composites. These agglomerates would then result in a weaker interfacial interaction between them and the polymer matrix due to having a lower surface area. Additionally, the irregular shape and size of the graphite flake agglomeration would decrease the efficiency of stress transfer from the matrix to the nanofillers. The random positions of the agglomerates could also act as points of defect within the matrix, further weakening the overall mechanical performance of the nanocomposite. 

On the other hand, the reason why nanocomposites incorporated with graphite flakes treated at 200 °C behaved differently to the other nanocomposites with treated flakes might be due to the higher temperature of the water treatment. This is because the higher the temperature of the water treatment, the higher the oxidation level of the carbon nanofillers [12]. However, while the higher temperature has resulted in improved functionalization, it has also caused structural damage to the nanofillers (as seen in the works of Zhang et al. [13]), which would explain why the tensile strength of the nanocomposites were not as strong as those with untreated flakes or flakes treated at 175 °C and 150 °C. Additionally, the damaged structure of the graphite flakes also affected the bonding between the nanofiller and the polymer matrix, which plays a significant role in the mechanical performance of the nanocomposite, which explains its similarity in tensile strength regardless of wt%, as all of the flakes have been affected by the high temperature of the water treatment. The decrease in tensile strength from 0.5 wt% to 2.5 wt% can be explained by the agglomeration of the increased nanofillers similar to the case for nanocomposites with flakes treated at 175 °C and 150 °C from 1.5 wt% onwards. The increase in tensile strength seen in 3.5 wt% and 4.5 wt%, however, can be explained by the increase in oxygen-containing functional groups on the flakes due to the increased functionalization caused by the higher temperature of the water treatment. This increase in oxidation level on the surface of the treated flakes has resulted in more bonding between them and the polymer matrix, which served to enhance the effectiveness of stress transfer across the nylon 610/graphite flake interface, which can overcome the effects of agglomeration of the nanofillers, leading to a higher tensile strength at a higher wt%. The bonds are formed between the oxygen functionalities on the surface of the treated flakes and the nylon molecules.

Moreover, the reason why nanocomposites added with untreated graphite flakes increases with increasing wt% is because the original flakes do not have any oxidative functional groups on its surface leading it to be more evenly distributed within the polymer matrix as there is less of a chance for the untreated flakes interacting with one another and forming aggregates at higher loading levels. Furthermore, at higher loading levels, the large number of flakes within the nylon 610 matrix will contribute a bridging effect in the polymer matrix, which will efficiently transfer the straining stress from the nylon 610 matrix to the nanofillers [14]. This same phenomenon is seen from 3.5 wt% to 4.5 wt% in nanocomposites with treated graphite flakes at 200 °C. However, the tensile strength of the nanocomposites with untreated flakes are at the lower end when compared to the rest of the other nanocomposites (with the exception of the one with 4.5 wt% flakes), as the oxygen containing functional groups on the treated flakes will form a stronger interfacial interaction between the nanofillers and the matrix, which will increase the efficiency of stress transfer when the nanocomposites are being elongated [15,16].

According to Figure 2, it can also be seen that the temperature of the water used during the supercritical water treatment of the nanofillers has an effect on the mechanical properties of the nanocomposites, as the tensile strength of the nanocomposites that incorporated the treated flakes increased from those treated at 200 °C to 175 °C and finally 150 °C. This is due to the increasing amount of damage caused by the higher temperature of the water treatment, as stated above. Thus, it is the composite with the nanofiller treated at the lowest temperature that has the highest tensile strength overall, as the graphite flakes have been functionalized by the water treatment, while not being sufficiently damaged by it. This indicates that there is a balance between the enhancements of the interfacial interactions between the functionalized graphite flakes and the nylon 610 matrix and the change to the mechanical structure of the treated flakes itself.

Another thing to note is that while there is a marked improvement seen in tensile strength for the nylon 610 nanocomposites when compared to that of neat nylon 610 (397.08 kPa), it is still lower than the tensile strength reported in other research regarding nylon nanocomposites. For example, the work of Moniruzzaman et al. [17] showed a 1 wt% single wall carbon nanotube/nylon 610 Composite that has a tensile strength of 79 MPa and a Young’s Modulus of 1.22 GPa. The reason for this is because the nylon 610 nanocomposites that were prepared in this research have not undergone any form of heat treatment, such as melt extrusion or melt blending, and were tested as is after synthesis via interfacial polymerization. This is also demonstrated further when the neat nylon 610 synthesized by other works also have higher tensile strength when compared to the neat nylon 610 produced in this research, as seen in Table 2 below. This is because one of the aims of this research was to report a quick and convenient method for the production of the nylon 610/graphite flake nanocomposites and, as a result, the nanocomposites seen in this work is not as sturdy as others. 

#### 3.1.2. Young’s Modulus

Figure 3 reveals a pattern where the Young’s Modulus for most of the nanocomposites increases with an increasing loading level of nanofiller, except for nanocomposites incorporated with graphite flakes treated at 150 °C and −0.08 MPa, where the modulus reaches a maximum at 1.5 wt% and proceeded to decrease afterwards. The reason for this increasing trend is because the addition of graphite flakes promoted the crystallization of nylon 610, as the nanofillers functioned as an excellent nucleation agent within the polymer matrix due to their small size (2–10 nm) and high aspect ratio [13,19]. Additionally, the increase in nanofillers could also result in an inter-blocking network of nanofillers in the nylon 610 matrix, which would give rise to the polymer chains being restricted, leading to higher rigidity behavior in the composites [20]. It would also seem that both the nucleation and inter-blocking effect have overcome the aggregation effect of the nanofillers at higher loading levels, as seen in Section 3.1.1. On the other hand, the decrease in Young’s Modulus after 1.5 wt% for composites with flakes treated at 150 °C can be attributed to the agglomeration of the increasing nanofillers, as it will weaken the inter-blocking effect of the flakes within the polymer matrix and reduce the interfacial interaction between the flakes and nylon molecules. As a result, the applied stress on the composite will be unable to be effectively transferred from the carbon nanofillers to the nylon 610 matrix [21]. This phenomenon will also result in the tensile strength of the nanocomposite incorporated with graphite flakes treated at 150 °C to decrease after 1.5 wt%, as seen in Figure 2. 

Similar to the above section, the Young’s Modulus of the nanocomposites incorporated with treated flakes decreased with the increase in the temperature of the water treatment. This is due to the modification to the surface morphology and the overall structure of the nanofillers as a result of the increasing temperature as stated in Section 3.1.1.

### 3.2. XRD Analysis

**Figure 4:**
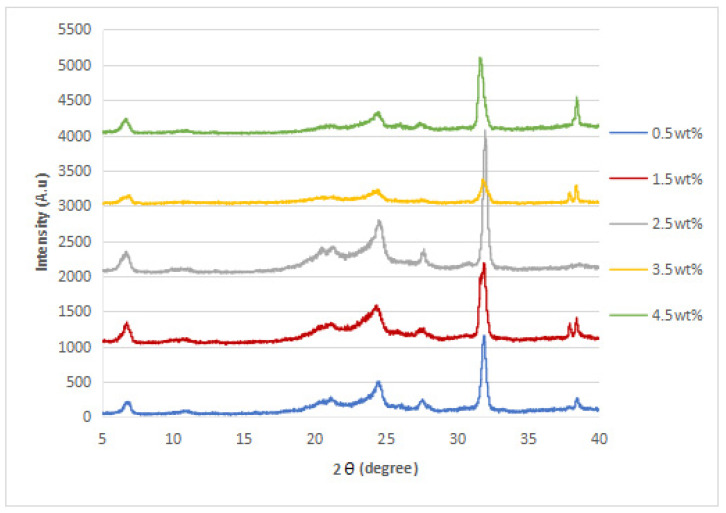
XRD Spectra of Nylon 610 Nanocomposites with Treated Graphite Flakes at 200 °C and −0.08 MPa.

**Figure 5:**
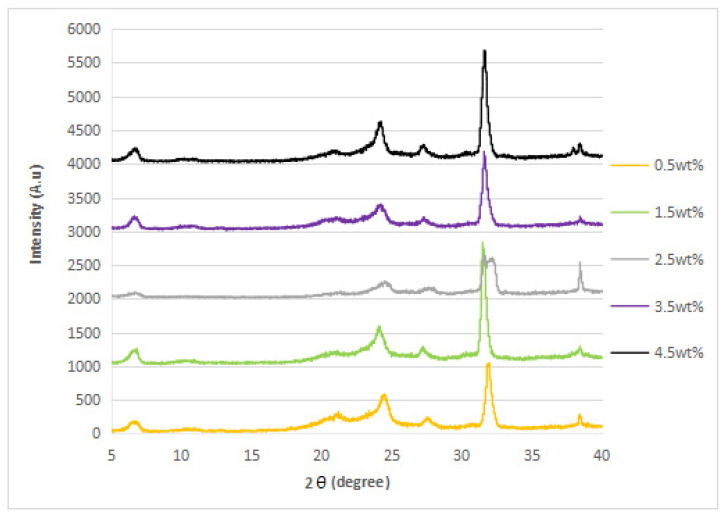
XRD Spectra of Nylon 610 Nanocomposites with Treated Graphite Flakes at 175 °C and −0.08 MPa.

**Figure 6:**
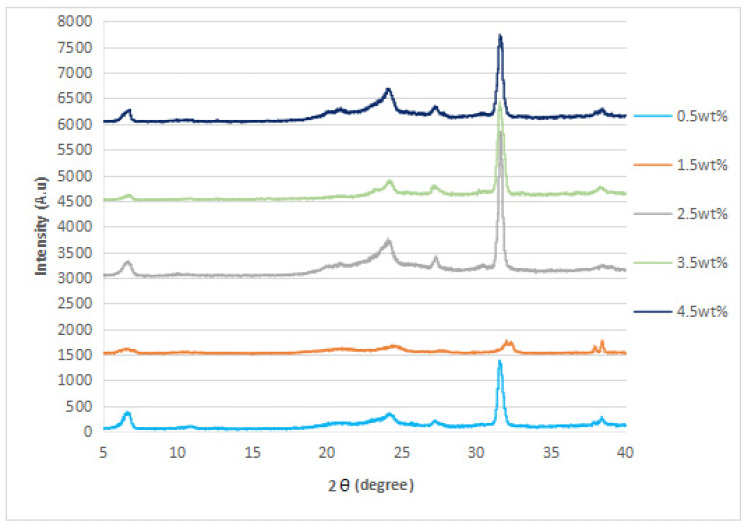
XRD Spectra of Nylon 610 Nanocomposites with Treated Graphite Flakes at 150 °C and −0.08 MPa.

**Figure 7:**
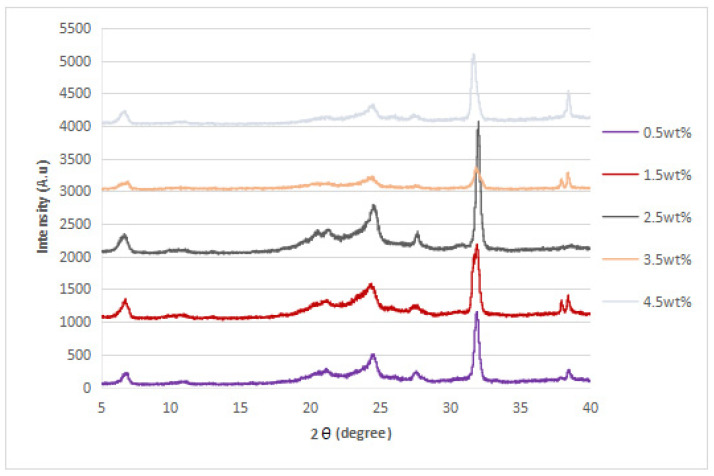
XRD Spectra of Nylon 610 Nanocomposites with Untreated Graphite Flakes.

**Figure 8:**
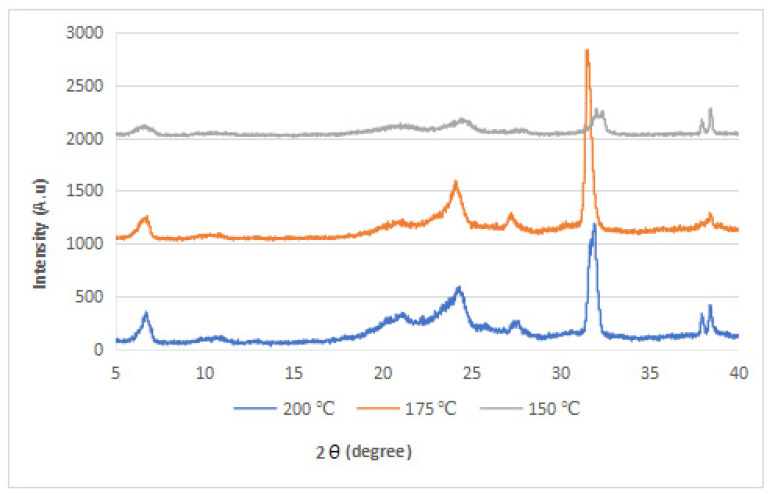
XRD Spectra for Nylon 610 Nanocomposites with 1.5 wt% Treated Graphite Flakes at All Temperatures for the Supercritical Water Treatment.

The XRD diffraction patterns of the nylon 610 nanocomposites that were incorporated with untreated or supercritical water-treated graphite flakes are displayed in Figure 4, Figure 5, Figure 6 and Figure 7; while Figure 8 displays XRD spectra of nylon 610 nanocomposites with 1.5 wt% treated flakes at all temperatures for supercritical water treatment. For all of the nanocomposites, a strong diffraction peak can be seen at 2θ = 24.1° and weak diffraction peaks arose at 2θ = 20.0° and 27.0°. The peaks at 2θ = 20.0° and 24.1° are characteristic peaks of the α-crystalline form of nylon 610, which correspond to the diffraction planes of (200) and (002/202) [22]. Additionally, the weak peak at 2θ = 27.0° represents the (001) plane of graphite [23]. Thus, it can be proven that the nanocomposites are made of two components, one of which is nylon 610 and the other is the graphite flakes. It is also shown in Figure 4, Figure 5, Figure 6 and Figure 7 that all of the nanocomposites possessed similar XRD spectra, with only the intensities of the peaks differing among one another. For instance, the peaks of nanocomposites added with flakes treated at 200 °C increased in intensity until it reaches a peak at 2.5 wt% before decreasing. The same situation is seen in nanocomposites with untreated flakes. However, for the nanocomposite incorporated with flakes treated at 150 °C and 175 °C, the peaks were shown to decrease in intensity until 1.5 wt% and 2.5 wt%, respectively, but then increase from that point on. As less intense peaks signify a more even dispersion of the nanofiller particles within the polymer matrix [24], this would suggest that the graphite flakes were more uniformly dispersed at lower wt% for nanocomposites with flakes treated at 150 °C and 175 °C, while the opposite for nanocomposites with untreated flakes and flakes treated at 200 °C was true. This observation is also in agreement with the tensile strength results seen in Section 3.1.1 (as uniform dispersion is one of the factors for high mechanical strength in composites) [15], as the tensile strength is higher at higher wt% for composites with untreated flakes and flakes treated at 200 °C and the reverse is true for composites incorporated with flakes treated at 150 °C and 175 °C. This is also supported by Figure 8, where the peaks of the composites with graphite flakes treated at 150 °C are significantly less intense than those with flakes treated at 175 °C and 200 °C.

While the XRD spectra of the nanocomposites are mostly similar to that of other nylon/graphite-based nanofiller nanocomposites, there are still some discrepancies that can be found. One example is the diffraction peak at around 2θ = 7.0°. In the research of graphene oxide reduction by Huang et al. [25], the peak at 10.0° corresponds to graphite after oxidation. One likely reason for the decrease to a lower angle might be because the oxidation of the graphite flakes by the water treatment was not thorough enough, as the temperature of the water is below supercritical condition, with the surface of the water-treated flakes being covered with fewer oxygen functionalities when compared to that of graphite oxide.

Additionally, the d-spacing and inter-chain separation for the deflection peak at 2θ = 24.1° in all of the nylon 610/graphite flake nanocomposites were tabulated in Table 3. As can be seen in the table below, before the amount of graphite flakes reaches 1.5 wt% or 2.5 wt%, an increase in the d-spacing and inter-chain separation for the peaks is shown, which denotes good dispersion of the flakes within the nylon 610 matrix [19]. After that point it decreases at higher wt%. The decrement in d-spacing and inter-chain separation at higher loading levels is accredited to the poor interaction between the flakes and nylon 610 matrix due to the agglomeration of the nanofillers. This is because the aggregation can result in a decrease in interlayer spacing between that of the graphite flake particles in the polymer matrix [26]. This agglomeration at higher wt% is in line with the SEM images of the nanocomposites in Section 3.4. This observation is seen in all of the nanocomposites, with either treated or untreated nanofillers.

**Figure 9:**
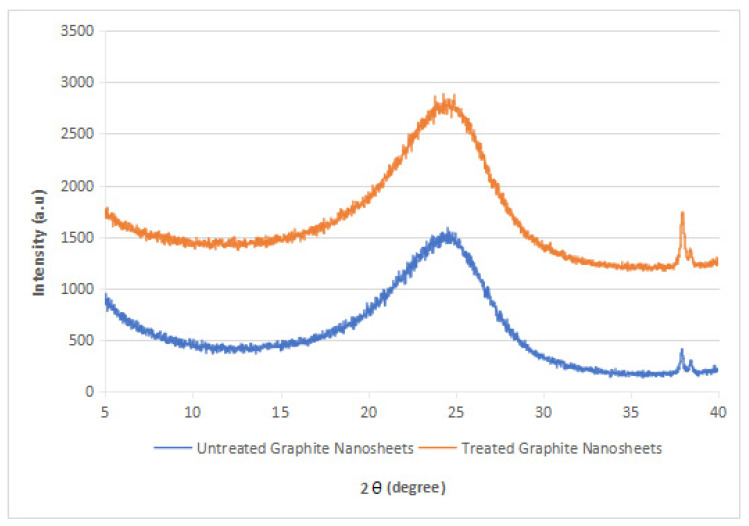
XRD Spectra of Treated and Untreated Graphite Flakes.

XRD spectra obtained from the graphite flakes before and after the supercritical water treatment are shown in Figure 9. Both the treated and untreated flakes displayed diffraction peaks at 2θ = 24.6°, which corresponds to the (001) plane of the graphite flakes. This result is also corroborated by Gupta et al. [27], who found that the XRD analysis of graphene oxide also showed similar results. The XRD apparatus has also determined that the d-spacing of the untreated graphite flakes is 3.6105 Å, while that of the treated flakes has increased to 3.6597 Å, as seen in Table 4. This is mainly because the water treatment has resulted in the water molecules being intercalated between the carbon sheets, along with the formation of oxygen functionalities on the surface of the graphite flakes, which led to the increase of the inter-chain separation. Moreover, the XRD diffraction pattern for the graphite flakes before and after water treatment did not change much, leading to the conclusion that supercritical water treatment did not alter the crystal structures of the nanofillers. The same phenomenon has also been noted by Wang et al. [15]. The intact crystal structure is expected to enhance the mechanical properties of the nanocomposites, which is seen in Figure 2, where nanocomposites with treated or untreated graphite flakes added have higher tensile strength than that of neat nylon 610, as carbon nanomaterials are known for their excellent mechanical properties [28]. However, there is also a difference between the XRD spectra of the graphite flakes and pure graphite. This is because the peak for the graphite diffraction plane has moved from 26.7° (as seen in virgin graphite) to 24.6°, which could be attributed to the presence of oxygen-containing functional groups on the graphite flakes. Additionally, the small peaks at around 38.0° for both graphite flakes both before and after water treatment could be due to the impurities found within the flakes.

### 3.3. FTIR Analysis

**Figure 10:**
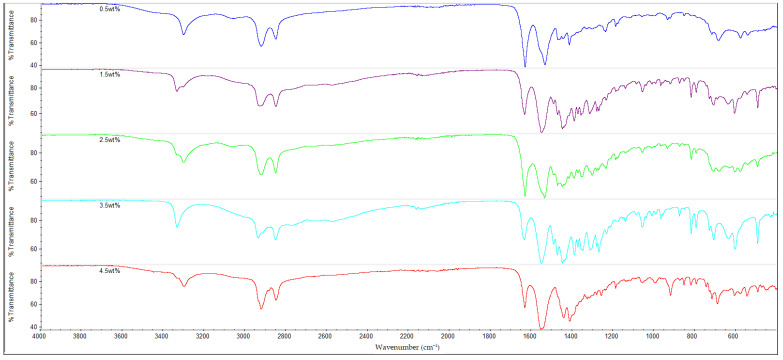
FTIR Spectra for Nylon 610 Nanocomposites with Treated Graphite Flakes at 200 °C and −0.08 MPa.

**Figure 11:**
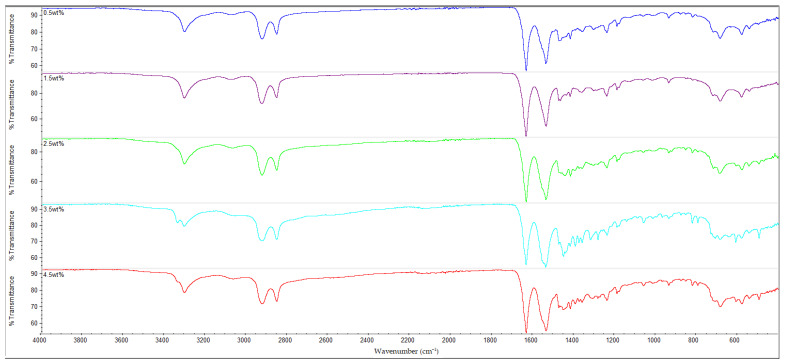
FTIR Spectra for Nylon 610 Nanocomposites with Treated Graphite Flakes at 175 °C and −0.08 MPa.

**Figure 12:**
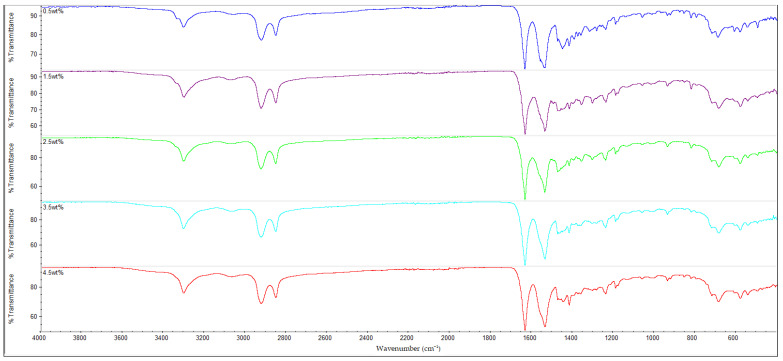
FTIR Spectra for Nylon 610 Nanocomposites with Treated Graphite Flakes at 150 °C and −0.08 MPa.

**Figure 13:**
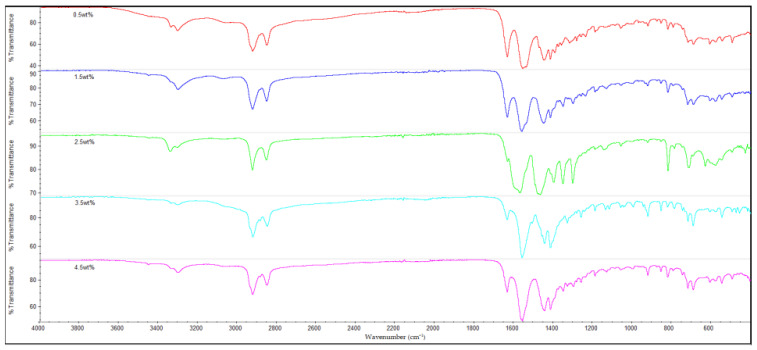
FTIR Spectra for Nylon 610 Nanocomposites with Untreated Graphite Flakes.

The results of the FTIR analysis for all of the samples are shown in Figure 10, Figure 11, Figure 12 and Figure 13. It is observable that all of the FTIR spectra below have close similarities to one another, along with showing the characteristic peaks of nylon 610. For instance, all of the nanocomposites possessed peaks at the region around 3300 cm^−1^, which correspond to the N-H stretching vibrations, indicating the presence of aliphatic primary amine; the peaks at 2925 cm^−1^ and 2850 cm^−1^, which are attributed to the stretching vibration of the C-H group within nylon 610 chain; the C=O stretching from the carbonyl groups and N-H stretching of amide I band at approximately 1635 cm^−1^; and the amide II band at approximately 1550 cm^−1^, which signifies the N-H deformation and C-N stretching [29,30]. As a result, it can be inferred that the inclusion of graphite flakes (treated or otherwise) did not significantly alter the chemical structure of nylon as the FTIR spectra of nylon 610/graphite flakes nanocomposites is similar to that of neat nylon 610. Moreover, the lack of significant differences between the nanocomposites incorporated with treated and untreated graphite flakes indicates that oxygen-containing functional groups on the treated flakes may not influence the structure of the nylon 610 matrix to a large degree.

**Figure 14:**
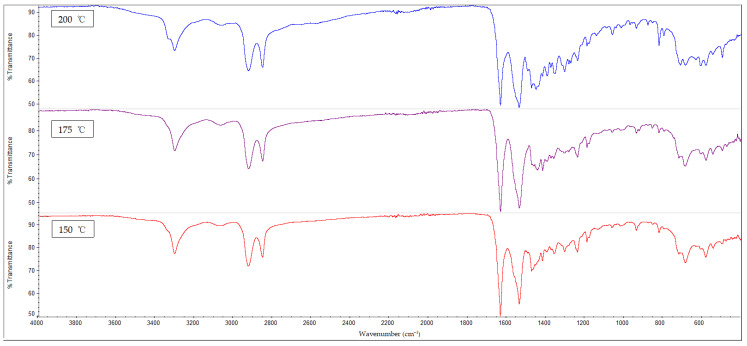
FTIR Spectra for Nylon 610 Nanocomposites with 2.5 wt% Treated Graphite Flakes at All Temperatures.

Additionally, the amide I band is comprised of two parts, ordered and disordered hydrogen-bonded carbonyl functional groups (CO-NH). The ordered groups composed the crystalline phases in the nylon 610 structure, while the disordered groups are associated with the amorphous domains [31]. Thus, the presence of this band indicates the formation of hydrogen bonding between the atoms of nylon 610 and the atoms of oxygen-containing functional groups on the graphite flakes, proving that the graphite flakes were successfully grafted onto the nylon 610 during the interfacial polymerization process. Besides that, the intensities of amide bands I, II and III (at peaks of 1635 cm^−1^, 1550 cm^−1^ and 1190 cm^−1^) are also indicative of the degree of hydrogen bonding between the nylon 610 and the nanofillers [32]. For example, when the amide peaks of the nanocomposites incorporated with untreated flakes at 1635 cm^−1^ and 1550 cm^−1^ were compared with that of nanocomposites with treated flakes, the intensities of their amide peaks were found to be slightly lower, which can be seen in the figures below. This is in line with the fact that supercritical water treatment is able to oxidize the surface of nanofillers, which will result in the rise of oxygen functionalities on the surface of the treated flakes. This will facilitate the formation of hydrogen bonds between the nanofillers and the nylon 610 molecules, which increases the efficiency of stress transfer when the nanocomposites are being elongated, thus increasing the tensile strength [15]. As a result, most nanocomposites incorporated with treated flakes have higher tensile strength than those incorporated with untreated flakes, as seen in Figure 2. Furthermore, FTIR spectra of nylon 610 nanocomposites incorporated with 2.5 wt% graphite flakes treated at all temperatures in supercritical water treatment are displayed in Figure 14. The figure shows a slight increase in intensity for amide II band (1550 cm^−1^) with the rise in water treatment temperature. Therefore, it could be stated that the increase in the temperature of supercritical water treatment will give rise to more oxygen-containing groups on the surface of graphite flakes.

As mentioned before, the FTIR peaks at 2925 cm^−1^ represent the C-H bonds in the nylon 610 polymer chain. By referring to Table 5, it is shown that the increase in loading levels of the graphite flakes (treated and untreated) will result in a slight increase the wavenumber of the C-H stretching peak, before decreasing at higher loading levels. For instance, for nanocomposites with nanofillers treated at 150 °C and −0.08 MPa, the wavenumber increases to a maximum at 2.5 wt% (2924.49 cm^−1^) before reducing to 2923.08 cm^−1^ at 4.5 wt%. This is because the non-polar nature of the nanofillers makes them capable of interacting well with the nylon 610 molecules (barring some oxygen-containing functional groups on the treated flakes), leading to the C-H bonds being strengthened [33], as the higher the wavenumber of an IR band, the stronger the bond. On the other hand, the decrease in wavenumber at higher wt% is due to the agglomeration of the nanofillers, as the increase in the number of flakes will amplify the probability of them aggregating together. This is due to the large aggregate particles having poor interaction with the polymer matrix, resulting in lower wavenumbers, as the original intermolecular interaction within the nylon 610 matrix has been diminished. This situation could also be seen in the drop in tensile strength at higher loading levels of treated graphite flakes due to agglomeration.

### 3.4. SEM Analysis

**Figure 15:**
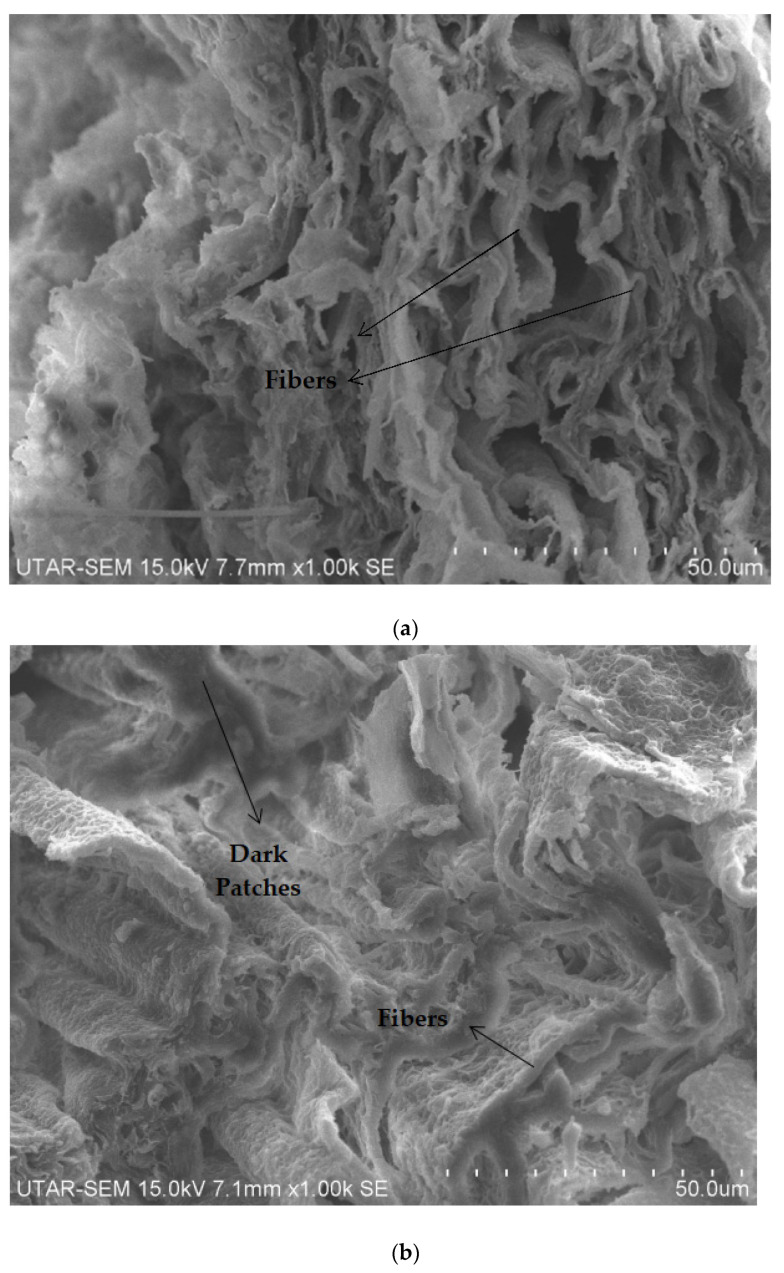

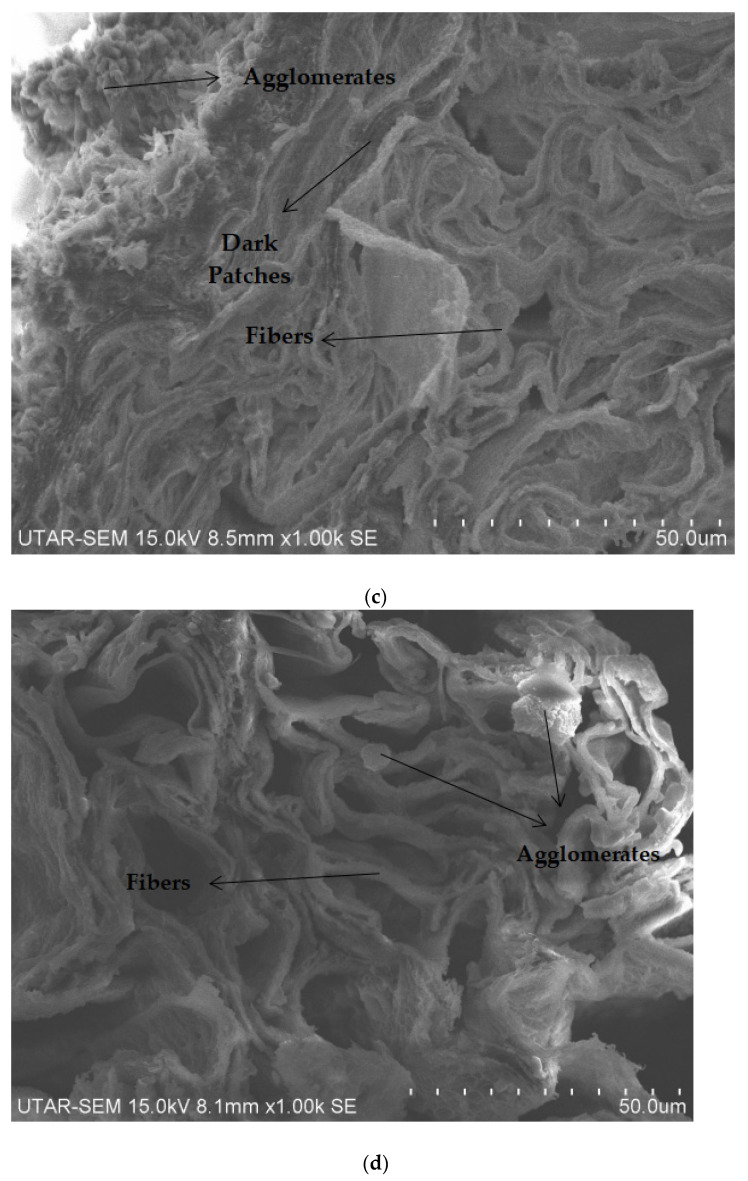

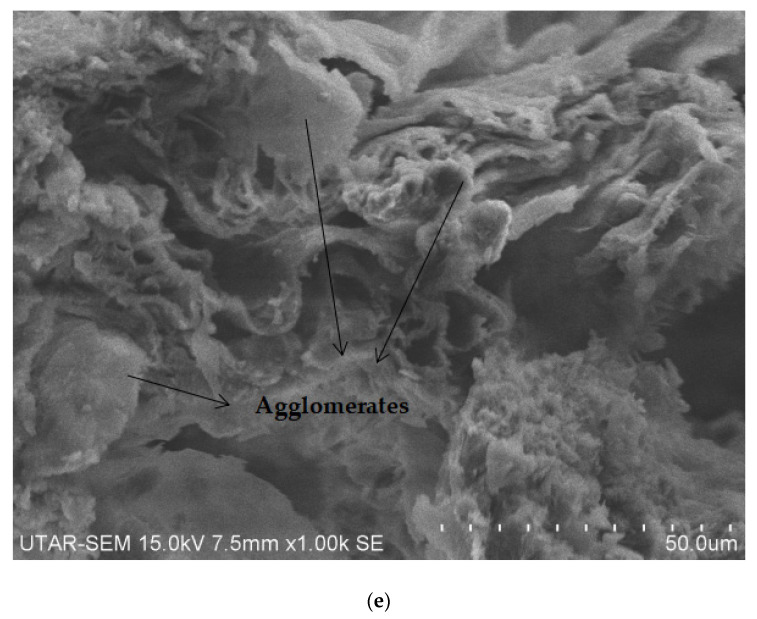
SEM Image of Nylon 610 Nanocomposites Added with Treated Graphite Flakes at 150 °C with (**a**) 0.5 wt%, (**b**) 1.5 wt%, (**c**) 2.5 wt%, (**d**) 3.5 wt%, (**e**) 4.5 wt% at × 1000 magnification.

Figure 15 below illustrates the fracture surface morphologies of all the nylon 610 nanocomposites incorporated with graphite flakes treated at 150 °C and −0.08 MPa at 0.5 wt% to 4.5 wt%. As can be seen in all of the SEM images, the nylon 610 nanocomposite structure is composed of interconnected fibers with a rough surface that have a worm-like shape. At lower loading levels of the nanofillers, the graphite flakes and nylon 610 were shown to have fine compatibility with one another, as the micrograph of the nanocomposite displayed a good matrix continuity, with the fibers having no obvious agglomeration or globule formation. This also suggest that the graphite flakes were evenly distributed within the polymer matrix, leading to a higher tensile strength [15]. However, as the wt% of the treated flakes increased, apparent agglomerations were observed on the surface of the fibers, especially at the higher wt%, as seen in Figure 15e. These agglomerates that surfaced on top of the nylon fibers can result in changes to the surface morphology of the nanocomposites, such as forming a stress concentration point during straining, which will lead to the overall weakening of mechanical properties. This phenomenon is in agreement with the results in Table 1, as seen in the reduction in tensile strength as wt% increases for nanocomposites incorporated with treated graphite flakes. 

Furthermore, with increasing loading levels of nanofillers, some of the nylon 610/graphite flake nanocomposite fibers have turned a darker color, as seen in Figure 15b onwards. This shows the influence of increasing graphite flakes. It is also noteworthy that even though there are observable effects of adding graphite flakes to the nylon 610 matrix, no obvious sign of graphite flakes could be found using SEM. The reason for this might be due to their relatively tiny number within the polymer matrix and its small size (2–10 nm). Is it possible that the individual carbon layers could only be seen using a device with higher magnification, such as a Transmission Electron Microscope (TEM).

The reason why only the nanocomposites with graphite flakes treated at 150 °C were shown as representative for the SEM images is because the fracture surface morphologies of all the nylon 610 nanocomposites are similar to one another, either incorporated with treated or untreated nanofillers. This is because all of them showed a network of rough fibers and the emergence of aggregations at higher loading levels of graphite flakes. Thus, it can be determined that the addition of graphite flakes will not considerably alter the surface morphology of the nylon 610 fibers.

## 4. Conclusions

In this study, it has been shown that nylon 610/graphite flake nanocomposites with tensile strength and Young’s Modulus that are several times larger than that of neat nylon 610 can be successfully synthesized by using an eco-friendly method of treating the nanofillers. This treatment is supercritical water treatment. Interfacial polymerization is also a convenient and less time-consuming method to create the nanocomposites, which will be useful when creating large quantities of the composites. Additionally, X-ray diffraction has revealed that the crystal structure of the treated and untreated graphite flakes is similar to one another, which is favorable when using them as nanofillers, as the original nature of the carbon nanomaterial can be retained, which will help improve the mechanical properties of the nanocomposites. FTIR spectra has also shown the presence of an amide I and II band, which is attributable to the hydrogen bonding between the graphite flakes and nylon 610 matrix. The intensity of the amide peaks for the nanocomposite with treated flakes is slightly higher than those incorporated with untreated flakes. The reason for this is the formation of oxygen-containing functional groups on the surface of the graphite flakes after water treatment, which will result in more bonds being formed between the nanofillers and the polymer matrix. 

Furthermore, the nanocomposites with treated flakes have lower tensile strength at a higher wt% due to the agglomeration of the nanofillers, while the opposite is true for nanocomposites with untreated flakes, as the lack of oxygen-containing groups on the surface of the untreated flakes minimizes interaction with one another at a high filler concentration. The Young’s modulus of all the nanocomposites generally increases with an increasing loading level due to the higher amount of nanofiller forming an inter-blocking network within the polymer matrix, leading to higher rigidity. Overall, supercritical water treatment of the nanofillers is beneficial to the mechanical properties of the polymer composite, as nanocomposites incorporated with treated flakes generally have higher tensile strength than those incorporated with untreated flakes. 

In summary, the enhancements to the mechanical properties for the nanocomposites can be credited to the intact crystal structure of the nanofillers and the interfacial interaction between the graphite flakes and the polymer matrix. The thermal effects of different temperatures used for supercritical water treatment on the carbon nanofillers have also been researched. It was determined that while the water treatment was able to form oxygen-containing functional groups on the surface of the graphite flakes, higher temperatures are also liable to cause some structural damage to the nanofillers, resulting in an overall deterioration in the mechanical properties of the nanocomposites. However, a good balance was found in the supercritical water treatment at 150 °C and −0.08 MPa between the functionalization of the graphite flakes and the structural integrity of the nanofiller. Therefore, it is concluded that the nylon 610 nanocomposite incorporated with 1.5 wt% graphite flakes treated at 150 °C and −0.08 MPa shows the highest potential for the development of mechanically stronger and tougher nanostructures. The relatively low temperature of the water treatment is also beneficial for saving energy while still being able to successfully functionalize the nanofillers. Another thing to note is that while the nanocomposite with 4.5 wt% untreated flakes has the second highest tensile strength, it is considered to be rather uneconomical, as the composite needs relatively large amounts of graphite flakes to synthesize.

## Figures and Tables

**Table 1:** Mechanical Properties of All the Nylon 610/Graphite Flake Nanocomposites.

Type of Nanofiller Incorporated in Nanocomposite	Loading Levels of Nanofiller (wt%)	Average Tensile Strength (kPa)	Average Young’s Modulus (MPa)
Treated Graphite Flakes at 200 °C and −0.08 MPa	0.5	603.14	50.431
	1.5	532.55	85.799
	2.5	518.18	54.442
	3.5	593.15	113.05
	4.5	640.76	123.67
Treated Graphite Flakes at 175 °C and −0.08 MPa	0.5	636.98	84.136
	1.5	654.05	88.105
	2.5	492.09	73.471
	3.5	616.763	96.827
	4.5	554.90	99.176
Treated Graphite Flakes at 150 °C and −0.08 MPa	0.5	690.87	118.96
	1.5	967.023	121.78
	2.5	724.27	89.467
	3.5	712.40	104.94
	4.5	760.30	116.42
Untreated Graphite Flakes	0	397.08	51.465
	0.5	323.54	42.934
	1.5	521.75	59.661
	2.5	531.81	111.18
	3.5	612.65	121.87
	4.5	930.50	137.44

**Table 2:** Comparison Between the Neat Nylon 610 Synthesized in This Research and in Other Research.

Neat Nylon 610 Synthesis Method	Average Tensile Strength (MPa)	Ref.
Interfacial Polymerization with No Added Heat Treatment	0.397	This Research
Interfacial Polymerization with Added Heat Treatment via Melt Extrusion	79	Moniruzzaman et al. [17]
Interfacial Polymerization with Added Heat Treatment via Hot Pressing	35.9	Kang et al. [18]

**Table 3:** d-spacing and Inter-chain Separation for All 2θ = 24.1° peaks in Nylon 610/Graphite Flakes Nanocomposites.

Type of Nanofiller Incorporated in Nanocomposite	Loading Levels of Nanofiller (wt%)	*d-*Spacing, Å	Inter-Chain Separation (R), Å
Treated Graphite Flakes at 200 °C and −0.08 MPa	0.5	3.64363	4.55868
	1.5	3.66284	4.58271
	2.5	3.70190	4.63158
	3.5	3.66284	4.58271
	4.5	3.66030	4.57954
Treated Graphite Flakes at 175 °C and −0.08 MPa	0.5	3.64510	4.56105
	1.5	3.68866	4.61502
	2.5	3.65247	4.56974
	3.5	3.67776	4.60138
	4.5	3.68372	4.60884
Treated Graphite Flakes at 150 °C and −0.08 MPa	0.5	3.67477	4.59764
	1.5	3.70190	4.63158
	2.5	3.68076	4.60514
	3.5	3.68001	4.60420
	4.5	3.68678	4.61266
Untreated Graphite Flakes	0.5	3.66284	4.58271
	1.5	3.69886	4.62778
	2.5	3.70190	4.63158
	3.5	3.64804	4.56420
	4.5	3.61592	4.52400

**Table 4:** d-Spacing and Crystallite Size for All 2θ = 24.6° Peaks in Treated and Untreated Graphite Flakes.

Sample	*d-*Spacing, Å	Inter-Chain Separation (R), Å
Treated Graphite Flakes	3.6597	4.5747
Untreated Graphite Flakes	3.6105	4.5132

**Table 5:** Wavenumbers of C-H Stretching Type for All of the Nylon 610/Graphite Flake Nanocomposites.

Type of Nanofiller Incorporated in Nanocomposite	Loading Level of Graphite Flakes, wt%	Wavenumber, cm^−1^
		C-H Stretching
Treated Graphite Flakes at 200 °C and −0.08 MPa	0.5	2922.93
	1.5	2923.69
	2.5	2926.52
	3.5	2938.26
	4.5	2923.34
Treated Graphite Flakes at 175 °C and −0.08 MPa	0.5	2922.90
	1.5	2924.25
	2.5	2923.42
	3.5	2923.38
	4.5	2922.80
Treated Graphite Flakes at 150 °C and −0.08 MPa	0.5	2923.18
	1.5	2923.65
	2.5	2924.49
	3.5	2923.50
	4.5	2923.08
Untreated Graphite Flakes	0.5	2924.18
	1.5	2924.04
	2.5	2924.76
	3.5	2924.11
	4.5	2923.53

## Data Availability

Not applicable.

## Supplementary Materials

The following supporting information can be downloaded at: https://www.mdpi.com/article/10.3390/polym14245494/s1, Figure S1: Average Tensile Strength of Nylon 610 Nanocomposites with Graphite Flakes Treated at 200 ℃ & −0.08 MPa; FigureS2: Average Tensile Strength of Nylon 610 Nanocomposites with Graphite Flakes Treated at 175 ℃ & −0.08 MPa; Figure S3: Average Tensile Strength of Nylon 610 Nanocomposites with Graphite Flakes Treated at 150 ℃ & −0.08 MPa; Figure S4: Average Tensile Strength of Nylon 610 Nanocomposites with Untreated Graphite Flakes; Figure S5: Average Young’s Modulus of Nylon 610 Nanocomposites with Graphite Flakes Treated at 200 ℃ & −0.08 MPa; Figure S6: Average Young’s Modulus of Nylon 610 Nanocomposites with Graphite Flakes Treated at 175 ℃ & −0.08 MPa; Figure S7: Average Young’s Modulus of Nylon 610 Nanocomposites with Graphite Flakes Treated at 150 ℃ & −0.08 MPa; Figure S8: Average Young’s Modulus of Nylon 610 Nanocomposites with Untreated Graphite Flakes.

## Abbreviations

XRD, X-ray diffraction; FTIR, Fourier transform infrared spectroscopy; SEM, scanning electron microscopy; CO_2_, carbon dioxide; HMDA, hexamethylenediamine; NaOH sodium hydroxide; TEM, Transmission Electron Microscope.
