# Supplementary material for: Study of Thermal Effect on the Mechanical Properties of Nylon 610 Nanocomposites with Graphite Flakes That Have Undergone Supercritical Water Treatment at Different Temperatures
Source: Polymers (Basel). 2022 Dec 15;14(24):5494. doi: 10.3390/polym14245494 (PMC9781813; doi:10.3390/polym14245494)
Supplement: Supplementary file 1 [file polymers-14-05494-s001.zip › polymers-2070173-supplementary.pdf]

Supplementary Materials

This section here illustrates the bar chart included with error bars for the tensile strength and Young's Modulus for all 21 of the nylon 610/graphite flakes polymer nanocomposites. The results shown in the bar charts below are exactly the same as in Figure 2 and 3 in the main research article.

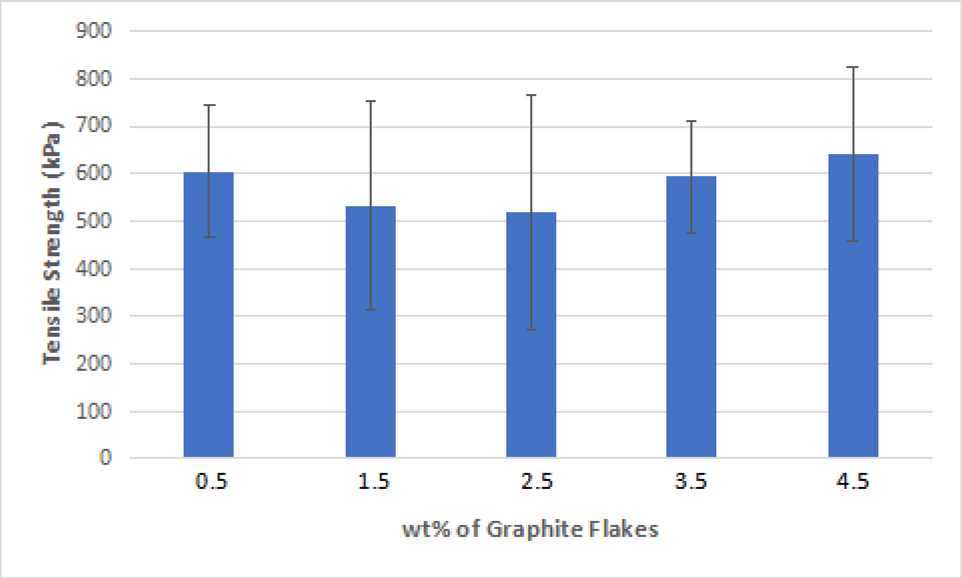

Figure S1: Average Tensile Strength of Nylon 610 Nanocomposites with Graphite Flakes Treated at 200 °C & −0.08 MPa

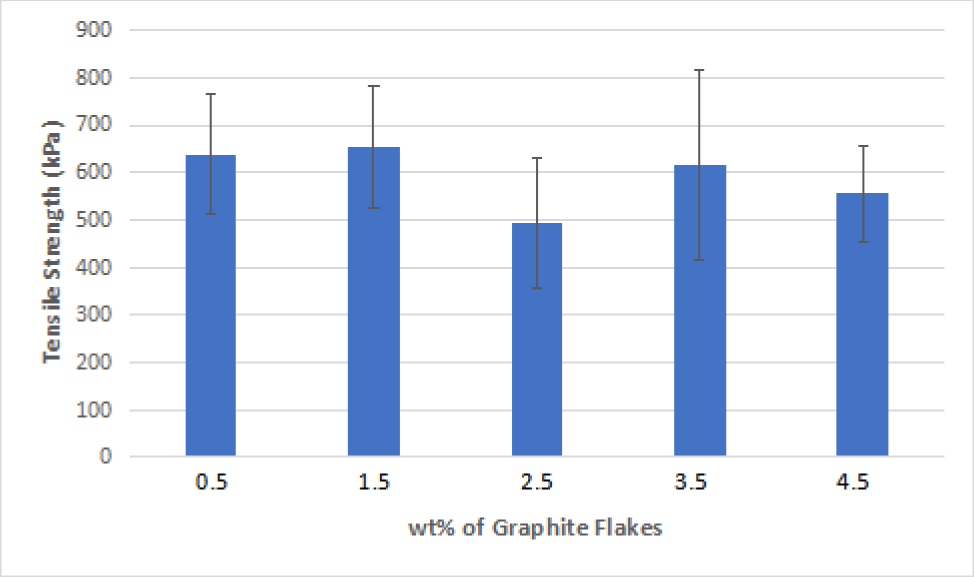

FigureS2: Average Tensile Strength of Nylon 610 Nanocomposites with Graphite Flakes Treated at 175 °C & −0.08 MPa

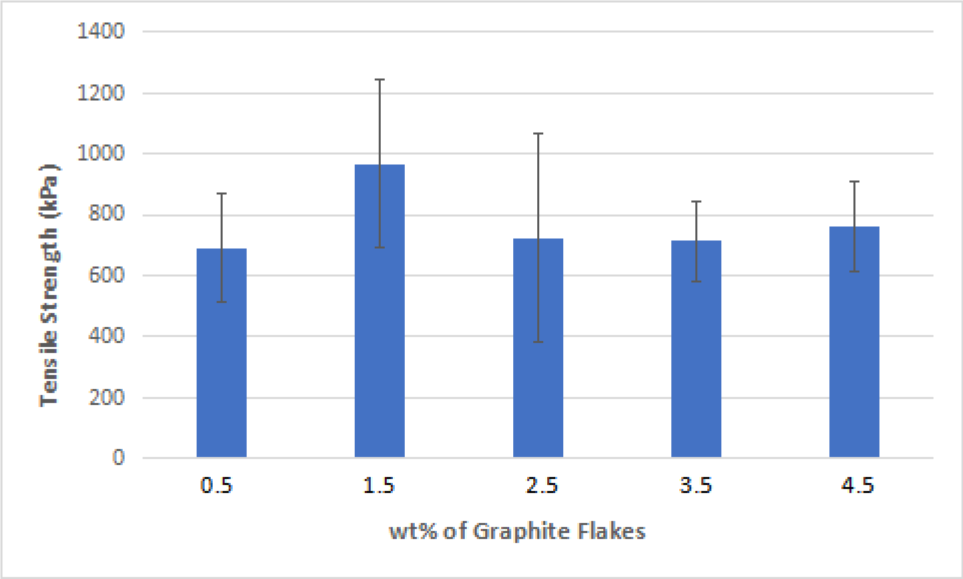

Figure S3: Average Tensile Strength of Nylon 610 Nanocomposites with Graphite Flakes Treated at 150 °C & −0.08 MPa

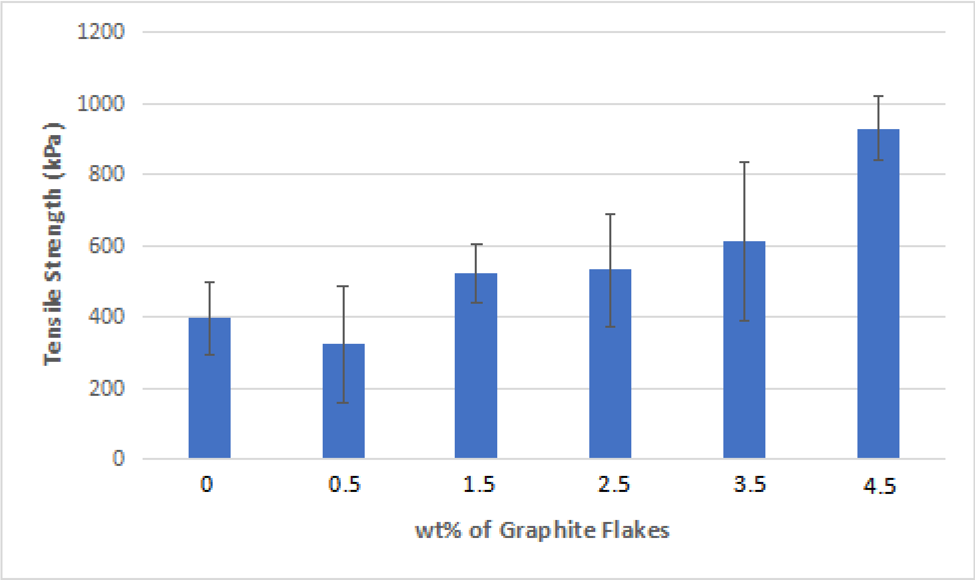

Figure S4: Average Tensile Strength of Nylon 610 Nanocomposites with Untreated Graphite Flakes

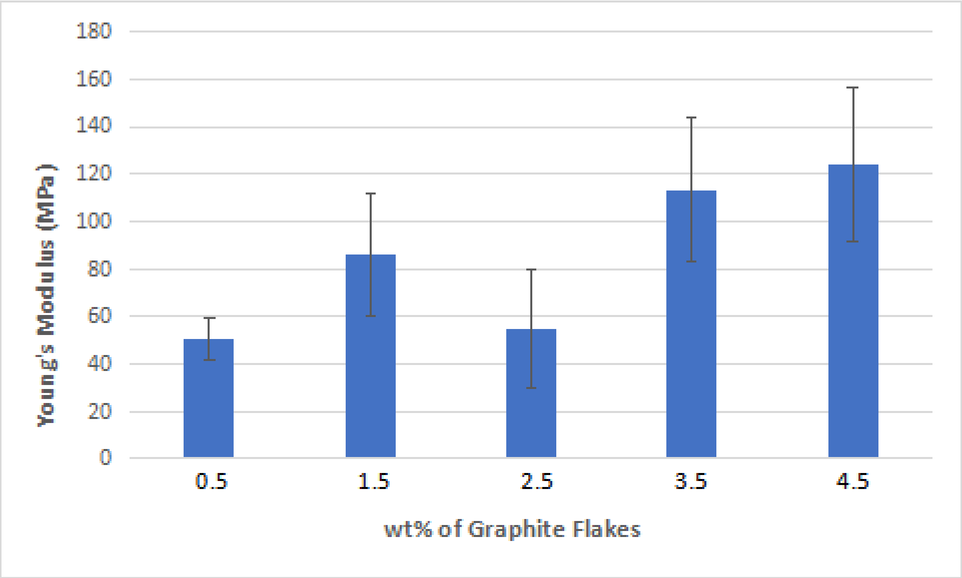

Figure S5: Average Young's Modulus of Nylon 610 Nanocomposites with Graphite Flakes Treated at 200 °C & −0.08 MPa

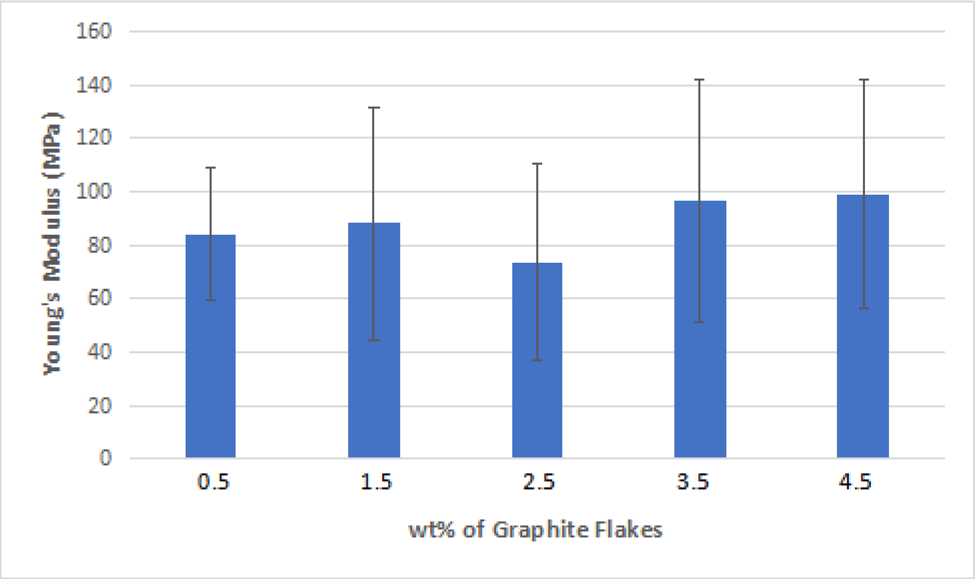

Figure S6: Average Young's Modulus of Nylon 610 Nanocomposites with Graphite Flakes Treated at 175 °C & −0.08 MPa

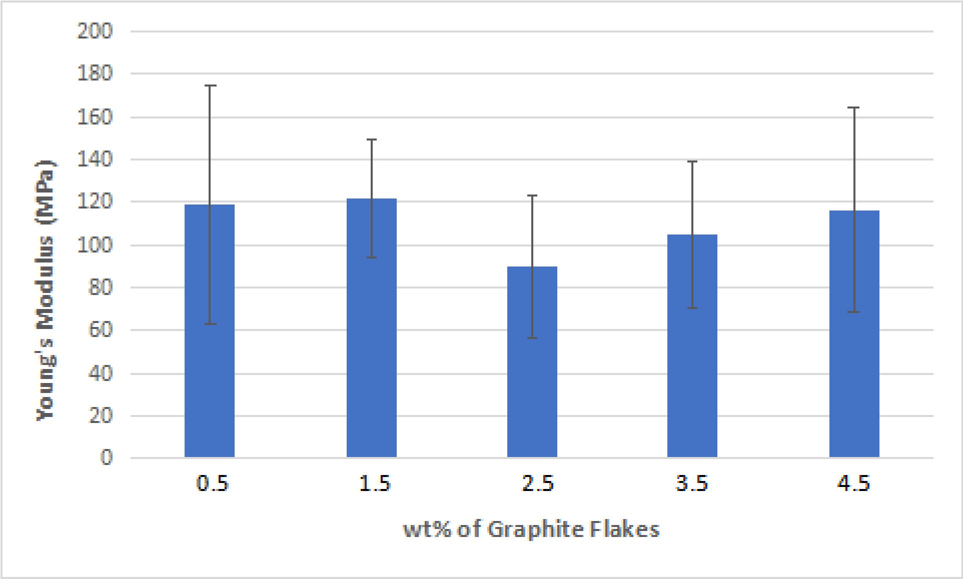

Figure S7: Average Young's Modulus of Nylon 610 Nanocomposites with Graphite Flakes Treated at 150 °C & −0.08 MPa

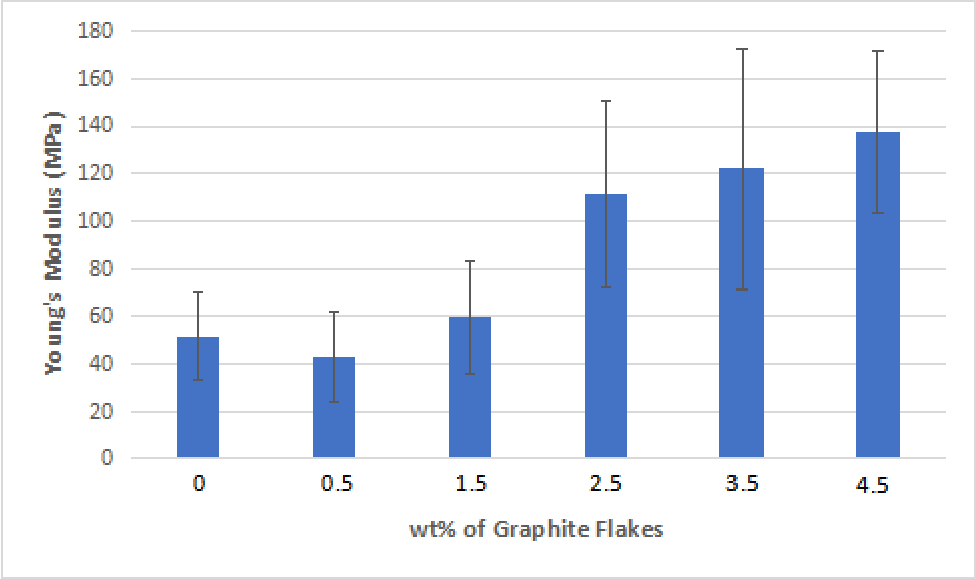

Figure S8: Average Young's Modulus of Nylon 610 Nanocomposites with Untreated Graphite Flakes
